# Povidone Iodine Disinfection Associated with Hypothyroidism and Potentially Contributing to Prolonged Kidney Failure

**DOI:** 10.1155/2021/5528210

**Published:** 2021-06-24

**Authors:** Yasmine Vercammen, Dieter Dauwe, Greet De Vlieger, Sabrina Houthoofd, Lars Desmet, Michael P. Casaer

**Affiliations:** ^1^Clinical Department of Intensive Care Medicine, UZ Leuven, Herestraat 49, 3000 Leuven, Belgium; ^2^Department of Cellular and Metabolic Medicine, KU Leuven, Herestraat 49, 3000 Leuven, Belgium; ^3^Department of Vascular Surgery, UZ Leuven, Herestraat 49, 3000 Leuven, Belgium

## Abstract

**Objectives:**

To report a case of povidone-iodine (PVP-I, Iso-Betadine®) disinfection of lower leg fasciotomy wounds resulting in iodide absorption and possibly contributing to hypothyroidism and prolonged kidney injury.

**Design:**

Case report. *Setting*. Pediatric intensive care unit (PICU), university hospital. *Patients*. A 13-year-old patient presenting with prolonged oligoanuric kidney failure and unexplained primary hypothyroidism three weeks after severe abdominal sepsis with multiple organ dysfunction and major rhabdomyolysis due to bilateral lower leg compartment syndrome, necessitating moderate size fasciotomies, disinfected daily with PVP-I. *Interventions*. Interruption of PVP-I exposure and initiation of thyroid hormone substitution. *Measurements and Main Results*. Hypothyroidism was revealed during diagnostic work-up for persistent hypertriglyceridemia. Thyroxine (T4) (4.0 mg/L) and tri-iodothyronine (T3) (64 ng/L) were moderately low, yet thyroid stimulating hormone (TSH) (16.8 mIU/L) was fourfold the maximal normal range value. This pattern, atypical for prolonged critical illness-related hypothyroidism, prompted interruption of PVP-I exposure and initiation of thyroid hormone substitution. Urinary production and creatinine clearance recovered during the following days, and one week later, intermittent renal replacement therapy could be terminated, suggesting that PVP-I toxicity and/or hypothyroidism may have contributed to the persistent renal failure three weeks after resolved septic shock and rhabdomyolysis. Elevated serum and urinary anion gap normalized simultaneously, but this evolution of rather nonspecific indices could be multifactorial.

**Conclusion:**

PVP-I is a commonly used broad-spectrum antimicrobial agent for prevention and treatment of wound infections. Toxic complications due to PVP-I absorption, after disinfection of extended thermal injuries larger than 20% of the body surface, have been described. In critically ill children, however, toxic effects of PVP-I may occur due to repeated disinfection of less extended wounds. Proposed screening strategies include: monitoring of the volumes of PVP-I applied daily; of the thyroid function, the serum, and/or urinary anion gap and the urinary iodide concentrations. These strategies, however, remain to be validated. This case report should be a wake-up call for daily integration of wound management in the clinical evaluation of critically ill patients.

## 1. Introduction

PVP-I (Iso-Betadine®) is a broad-spectrum antimicrobial agent commonly used for prevention and treatment of wound colonization and infections. It is a chemical complex of iodine with microbicidal activity and polyvinylpyrrolidone (povidone), a synthetic polymer. The antiseptic is widely used as a 10% solution in water, containing 1% iodine [1, 2]. Despite its potential beneficial effects on wound healing [3], there are several reports published on metabolic and toxic complications due to absorption of the therapeutic agent [4, 5], especially after disinfection of thermal injuries larger than 20% of the body surface [6].

We here present the atypical case of PVP-I disinfection of moderate size fasciotomy wounds resulting in unexpected intoxication and hypothyroidism and possibly contributing to delayed recovery of acute kidney failure in the context of resolved septic shock.

## 2. Case Report

We present the case of a 13-year-old boy who was hospitalized at the PICU of the University Hospitals Leuven after acute laparotomy for small bowel obstruction resulting in an ischemic bowel and a 30 cm enterectomy. Postoperatively, the patient suffered severe septic shock with acute septic cardiomyopathy and multiple organ failure including liver failure and disseminated intravascular coagulation (DIC). Within hours, he developed a compartment syndrome of the lower extremities and concurrent rhabdomyolysis with a level of creatine kinase (CK) reaching to 250000 U/L (reference ≤190 U/L), requiring urgent medial and lateral fasciotomy of both lower legs on ICU day 1. CK levels declined promptly to 40000 U/L on ICU day 3, 4834 U/L on ICU day 6, and 351 U/L by ICU day 7. Acute kidney injury (AKI) initially required continuous venovenous hemofiltration (CVVH) and, after 10 days, intermittent hemodialysis (IHD) therapy. Fasciotomy wounds were initially marginally perfused with important edema of the lower legs and feet, necessitating postponing the closure of the fasciotomy wounds until after a month. In order to prevent infection in these high-risk wounds, the subcutaneous tissue and the surrounding skin was disinfected daily with antiseptic PVP-I solution at a concentration of 10%. Wound cultures remained sterile during the entire open wound treatment interval, and from ICU day 12 onward, daily wound care was skipped on several occasions allowing the patient some rest, given the good clinical wound evolution (see Table 1).

**Figure 1:**
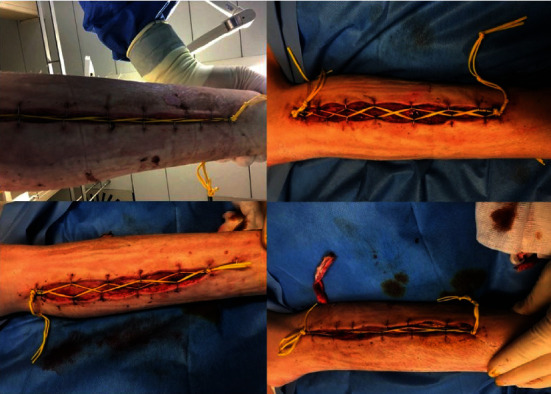
Fasciotomy wounds on both sides of both lower legs.

**Figure 2:**
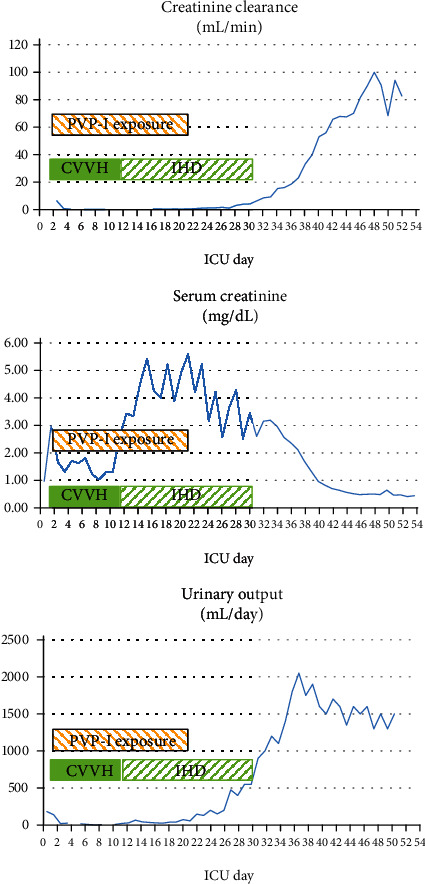
Spontaneous recovery of measured creatinine clearance, normalization of serum creatinine, and urinary output in the days following interruption of PVP-I exposure on ICU day 21. CVVH: continuous venovenous hemofiltration; IHD: intermittent hemodialysis; PVP-I: povidone-iodine disinfection.

Septic shock, liver failure, and DIC resolved within a week, and the patient was weaned from the ventilator by day 14. Acute kidney injury however did not recover (see Table 2), and triglycerides were rising without apparent reason, contributing potentially to acute pancreatitis. Diagnostic work-up revealed plasma TSH levels at day 23 that were highly increased to 16.8 mU/L (reference range 0.27-4.20 mU/L), levels of T4 4.0 mg/L (reference range 5.1 -14.1) and T3 64 ng/dL (reference range 80-200 ng/dL) was diminished, whereas reverse T3 was slightly increased, and reverse T3 was slightly increased (42 ng/dL). There was a high suspicion of hypothyroidism secondary to absorption of povidone iodine after 3 weeks of extensive wound care (see wound pictures taken on day 22 in Figure 1) with PVP-I. Significant systemic iodine absorption was documented by very high levels of urinary iodine excretion (>10,000 *μ*g/L). Disinfection with PVP-I was formally stopped at ICU day 23 (last application on ICU day 21) and thyroid hormone suppletion was initiated. There was an elevated urinary anion gap of 42.2 mmol/L. At days 29 and 31, urinary iodine level was at a toxic concentration of more than 10,000 *μ*g/L with a urinary anion gap of 40.4 mmol/L. Once the exposure was interrupted, urinary iodine values gradually decreased and urinary output and measured creatine clearance increased as soon as 4 days later, in the absence of diuretics and with increasing urinary creatinine excretion (Tables 2 and 3 and Figure 2). IHD could be interrupted at day 30.

Thyroid hormone (intravenous thyroxin-L-sodium, initially started at 100 *μ*g daily and reduced to 50 *μ*g daily after 3 days) was substituted from day 25 until day 45; thereafter, the thyroid function recovered. The fasciotomy wounds on the left side were closed on ICU day 30. The fasciotomy on the right leg could not be approximated because of excessive of traction and healed spontaneously after one month. Finally, at day 54, the patient could be discharged from the intensive care unit. The patient was evaluated 4 months later in outpatient pediatric clinic and was doing very well; kidney function and renal ultrasound were reassuring.

## 3. Discussion

PVP-I is considered a very safe and effective topical antiseptic. However, cases of systemic toxicity have been reported before. As described by Pietsch and Meakins [6], disinfection of extensive thermal injuries with PVP-I has occasionally been associated with acidosis, thyroid dysfunction, and renal failure, due to absorption of the therapeutic agent [6, 7]. In this case, PVP-I absorption possibly has aggravated sepsis-induced AKI and acidosis. Iodine and povidone can both be systemically absorbed, and it is still uncertain whether iodine or rather the acidic complex povidone-iodine (pH = 2.43) causes the different toxic effects [6, 7]. Pietsch and Meakins [6] recommend against the topical use of PVP-I in thermal injuries greater than 20% body surface or in the presence of renal failure, as absorption of the agent may be enhanced in nonintact skin and greater wound areas [7]. In this report, we describe a case of systemic toxicity after disinfection of less extended wounds after fasciotomy. Toxicity manifestations were a combination of metabolic and electrolyte abnormalities, renal impairment, and thyroid dysfunction. Cases of thyroid dysfunction induced by PVP-I have been reported before [8]. This phenomenon may be induced by the Wolff-Chaikoff effect, as elevated iodine plasma levels temporally block the synthesis of thyroid hormones [9]. In particular, newborns and patients with underlying thyroid disorders seem to be more prone to this toxicity [8, 9].

Besides thyroid dysfunction, blood tests revealed metabolic and electrolyte abnormalities such as high anion gap metabolic acidosis, hyperchloremia, and elevated urinary anion gap. In most cases, it is difficult to reveal the cause of metabolic acidosis because of concurrent disorders [6, 10]. In this patient, metabolic acidosis could have been the result of septic shock, MODS, and AKI (Table 3). Typically, serum chloride levels could be falsely high, because of the interference by iodine, a “chloride look-alike” halogen, resulting in an overestimation of the actual chloride amount [10–12]. Iodine is renally excreted by glomerular filtration; consequently, systemic accumulation of iodine is seen more often in patients with renal impairment [13]. Filtered iodine molecules may have direct toxic effects on the tubular cells, as two patients with AKI and povidone-iodine intoxication had necrosis of tubular cells and interstitial nephritis on kidney biopsy [13, 14]. Although we did not perform a kidney biopsy in our patient, the favorable evolution of the renal function upon discontinuation of PVP-I application suggests that iodine toxicity was possibly a contributing factor to kidney injury and delayed recovery. In addition, the elevated urine anion gap often reflects a renal acidification defect [15], which may possibly result from renal toxicity of iodine.

Besides the evidence of direct toxic effect of iodine molecules, we should also consider indirect effects on renal function. Hypothyroidy can be associated with acute and chronic kidney disease, with improved renal function upon thyroid hormone supplementation [16–18]. The initiation of thyroid hormones maybe an additional contributing factor to enhanced recovery of kidney function in this case. Finally, we cannot exclude that the recovery of renal function shortly after interruption of PVP-I exposure, and initiation of thyroid hormone was a chance finding, given the uncontrolled nature of the data presented. Estimation of the true contribution of both PVP-I exposure and hypothyroidism to occurrence and persistence of AKI would require comparative studies such as randomized animal experiments.

The value of an elevated urinary anion gap, unexplained hypothyroidism, and hyperchloremia as efficacious parameters to screen for PVP-I toxicity needs to be evaluated in prospective studies. For the time being, we recommend to consider potential iodine intoxication in all critically ill patients treated for several days with PVP-I, particularly when AKI persists or when unexplained hypothyroidism occurs. After weighing the potential systemic/toxic impact of PVP-I disinfection against the extent of wound infection or bacterial colonization, the treating multidisciplinary team should consider substituting PVP-I disinfection with other antiseptics, such as chlorhexidine (with or without cetrimide) and/or substitution PVP-I topical ointment with, for example, silver-based ointments (such as silver sulfadiazine cream).

*Patient perspective*: prior to sending the informed consent form, we contacted the parents of the patient by phone, more than six months after ICU discharge. The child was doing very well and enjoyed going back to school, but strength in the lower part of the legs was still suboptimal as compared to the premorbid status.

## 4. Conclusion

The potential for toxic effects after PVP-I disinfection of moderate size wounds might be underestimated and thus go unrecognized. Timely recognition of iodine toxicity during the topical PVP-I exposure and awareness of its potentially severe complications may contribute to optimal management and a favorable outcome. More generally, this case report pleads for daily integration of wound evolution and wound management in the clinical evaluation of critically ill patients.

## Figures and Tables

**Table 1:** Evolution from ICU day 0 until day 54 of serum levels of TSH, T3, T4, and TBG and of urinary iodine concentration. Wound care applied on these ICU days.

ICU day	TSH (mIU/L)	T3 total (ng/dL)	T4 total (mg/dL)	TBG (mg/L)	Urinary iodine (mg/L)	Wound care fasciotomy wounds
Reference	0.27-4.20	80-200	5.1-14.1	14.0-24.0	≤100	
0						
1						
2						NaCl 0.9%+Iso-Betadine® gel
3						NaCl 0.9%
4						
5						
6						NaCl 0.9%+Iso-Betadine® tulle
7						
8						
9						NaCl 0.9%+Dakin Cooper®+Iso-Betadine® tulle
10						NaCl 0.9%+Dakin Cooper®
11						NaCl 0.9%+Dakin Cooper®+Iso-Betadine® tulle
12						NaCl 0.9%+Dakin Cooper®+Iso-Betadine® tulle
13						
14						NaCl 0.9%+Dakin Cooper®+Iso-Betadine® dressing
15						NaCl 0.9%+Braunol® 7.5%
16						NaCl 0.9%+Dakin Cooper®
17						NaCl 0.9%
18						NaCl 0.9%
19						
20						
21						NaCl 0.9%+Braunol® 7.5%
22						
23	16.8	64	4.0	22.1		
24						NaCl 0.9%
25	13.3	59	3.9	20.6		
26						
27						
28	5.35	44	6.4	23.8		
29					>10000	NaCl 0.9%+chlorhexidine digluconate 0.5% in ethanol 70%+Flaminal Forte®
30						
31					>10000	
32						NaCl 0.9%+Flamigel®
33						
34						
35	5.03	59	4.5	19.7		
36						NaCl 0.9%+Flaminal Forte®
37						
38					>10000	NaCL 0.9%+Dakin Cooper®
39						
40						
41						NaCl 0.9%
42	3.51	82	7.8	19.0		
43						
44					3413	NaCl 0.9%+Flaminal Forte®
45						NaCl 0.9%+Flaminal Forte®
46						NaCl 0.9%+Flaminal Forte®
47						NaCl 0.9%
48						NaCl 0.9%+Flaminal Forte®
49	4.28	71	5.3	17.2		NaCl 0.9%+Flaminal Forte®
50						NaCl 0.9%+Flaminal Forte®
51						NaCl 0.9%+chlorhexidine digluconate 0.5% in ethanol 70%+Flaminal Forte
52						NaCl 0.9%+Flaminal Forte®
53	7.82					
54						

Abbreviations: T3: tri-iodothyronine; T4: thyroxine; TSH: thyroid stimulating hormone; TBG: thyroid binding globulin.

**Table 2:** Evolution from ICU day 0 until 54 of measured creatinine clearance, serum creatinine, 24 h urine output volumes, and urinary iodine concentrations during ICU stay.

ICU day	Creatinine clearance, BSA corrected (mL/min)	Creatinine (mg/dL)	Diuresis 24 h (mL)	Urinary iodine (mg/L)
Reference		0.46-0.77		≤100
0		0.97	182	
1	CVVH	Start CVVH	140	
2	CVVH	CVVH	20	
3	CVVH	CVVH	25	
4	CVVH	CVVH		
5	CVVH	CVVH	17	
6	CVVH	CVVH	8	
7	CVVH	CVVH	2	
8	CVVH	CVVH	4	
9	CVVH	CVVH		
10	CVVH on hold	CVVH on hold	8	
11	CVVH on hold	CVVH on hold	22	
12	IHD	IHD	30	
13			67	
14	IHD	IHD	44	
15			35	
16	IHD	IHD	30	
17			25	
18			40	
19	IHD	IHD	42	
20			74	
21	IHD	IHD	56	
22			147	
23	IHD	IHD	130	
24			200	
25	IHD	IHD	150	
26			200	
27			475	
28	IHD	IHD	400	
29			550	>10000
30	IHD	IHD	550	
31	6.44	2.60	900	>10000
32	8.6	3.15	1000	
33	9.22	3.19	1200	
34	15.3	2.96	1100	
35	16.07	2.56	1400	
36	18.6	2.36	1800	
37	23.04	2.12	2050	
38	33.11	1.68	1750	>10000
39	39.75	1.32	1900	
40	53.02	0.96	1600	
41	55.83	0.82	1500	
42	65.71	0.70	1700	
43	67.83	0.64	1600	
44	67.52	0.57	1350	3413
45	44.67	0.52	1600	
46	81.7	0.48	1500	
47	90.03	0.49	1600	
48	99.95	0.50	1300	
49	90.62	0.48	1500	
50	68.30	0.64	1300	
51	94.12	0.46	1500	
52	82.71	0.47		
53		0.41		
54		0.44		

**Table 3:** Evolution from ICU day 0 until day 54 of serum anion gap and electrolytes, arterial lactate, urine iodine concentration, anion gap, and electrolytes.

ICU day	Serum			Arterial	Urinary				
	Anion gap (mEq/L)	Cl^−^ (mmol/L)	HCO_3_^−^ (mmol/L)	Lactate (mmol/L)	Iodine (mg/L)	Anion gap (mEq/L)	Cl^−^ (mmol/L)	K^+^ (mmol/L)	Na^+^ (mmol/L)
Reference	Sep-20	98.0-107.0	22.0-29.0	0.5-2.2	≤100				
0	30.7	101.2	14.2	8.9					
1	34.7	98.7	15.7	3.6					
2	19.7	102.3	21.9	3.5					
3	18.1	102.6	21.6	2.5					
4	20.4	101.9	20.5	3.6					
5	19.0	100.9	21.6	2.2					
6	17.5	102.9	22.3	1.0					
7	15.5	104.6	21.6	0.8					
8	18.2	101.6	20.3	0.9					
9	20.5	96.2	22.8	0.8					
10	19.9	97.0	24.1	0.9					
11	19.1	96.1	22.6	1.3					
12	20.8	96.8	18.5	0.8					
13	23.8	97.0	16.2	0.8					
14	27.2	93.4	18.1	0.6					
15	33.3	88.4	21.3	0.7					
16	25.5	93.5	25.1	1.0					
17	22.3	95.5	27.7	1.0					
18	23.6	91.7	29.8	0.8					
19	21.0	93.3	21.5	0.8					
20	27.0	92.3	19.6	1.0					
21	28.1	88.7	22.2	1.0					
22	25.2	94.2	19.7	0.7					
23	28.1	91.5	19.0	0.7					
24	23.3	99.2	17.5	0.7		42.2	53	28	67.2
25	24.1	95.4	19.9	1.0					
26	20.2	102.5	17.7	0.7					
27	20.2	98.7	21.0	0.8					
28	24.0	96.6	23.7	0.7					
29	21.3	101.6	19.2	1.0	>10000				
30	22.6	102.2	24.6	1.6					
31	19.9	104.3	22.1	0.9	>10000	40.4	54	24	70.4
32	21.3	101.1	25.7	0.9					
33	22.7	102.0	28	0.8					
34	20.8	102.7	25.8	0.7					
35	21.2	109.4	21.2	0.7					
36	22.2	110.8	16.7	0.8					
37	22.2	113.1	16.1	0.8					
38	15.6	123.9	16.8		>10000				
39	17.8	116.1	17.1						
40	19.6	112.1	16.8						
41	18.7	113.1	15.0						
42	14.2	111.7	17.6						
43	15.5	107.6	18.6						
44	19.7	104.0	16.0		3413				
45	19.3	104.6	17.5						
46	15.9	105.4	18.9						
47	17.6	105.3	19.5						
48	21.2	98.3	18.4						
49	22.1	98.9	19.6			20.4	26	24	22.4
50	18.7	102.9	21.4						
51	16.3	101.2	24.2						
52	16.3	99.1	26.9						
53	15.9	102.1	25.5						
54	16.1	103.8	23.7						

## Data Availability

The underlying data are part of the patient's medical file and have been consulted with permission of the patient's parents.
